# A narrative review on the role of magnesium in immune regulation, inflammation, infectious diseases, and cancer

**DOI:** 10.1186/s41043-023-00423-0

**Published:** 2023-07-27

**Authors:** Sumel Ashique, Shubneesh Kumar, Afzal Hussain, Neeraj Mishra, Ashish Garg, B. H. Jaswanth Gowda, Arshad Farid, Gaurav Gupta, Kamal Dua, Farzad Taghizadeh-Hesary

**Affiliations:** 1 Department of Pharmaceutics, Pandaveswar School of Pharmacy, Pandaveswar, West Bengal 713378 India; 2 Department of Pharmaceutics, School of Pharmacy, Bharat Institute of Technology (BIT), Meerut, 250103 UP India; 3 Department of Pharmaceutics, College of Pharmacy, King Saud University, 11451 Riyadh, Saudi Arabia; 4 Department of Pharmaceutics, Amity Institute of Pharmacy, Amity University Madhya Pradesh (AUMP), Gwalior, MP 474005 India; 5 Department of Pharmaceutics, Guru Ramdas Khalsa Institute of Science and Technology (Pharmacy), Jabalpur, Madhya Pradesh India; 6 Department of Pharmaceutics, Yenepoya Pharmacy College & Research Centre, Yenepoya (Deemed to Be University), Mangalore, 575018 India; 7 Gomal Center of Biochemistry and Biotechnology, Gomal University, D.I.Khan, KPK Pakistan; 8 School of Pharmacy, Suresh Gyan Vihar University, Mahal Road, Jagatpura, Jaipur India; 9 Discipline of Pharmacy, Graduate School of Health, University of Technology Sydney, Ultimo, NSW 2007 Australia; 10 Faculty of Health, Australian Research Centre in Complementary and Integrative Medicine, University of Technology Sydney, Ultimo, Australia; 11 ENT and Head and Neck Research Center and Department, The Five Senses Health Institute, School of Medicine, Iran University of Medical Sciences, Tehran, Iran; 12 Department of Clinical Oncology, Iran University of Medical Sciences, Tehran, Iran

**Keywords:** Magnesium, Cancer, Immune modulator, Infectious diseases

## Abstract

**Background:**

Magnesium (Mg) has gained much importance recently because of its unique range of biological functions. It is one of the most significant micronutrients in biological systems. This review aims to outline the immune-regulating actions of Mg and its crucial role in regulating inflammation and immune response to infectious agents and malignancies.

**Methods:**

We conducted a literature review on MEDLINE, PubMed, EMBASE, Web of Science to determine the impact of Mg on immune regulation in three settings of inflammation, infection, and cancer. We thoroughly examined all abstracts and full-text articles and selected the most relevant ones for inclusion in this review.

**Results:**

Mg has long been associated with immunological responses, both nonspecific and specific. It plays a pivotal role in diverse immune responses by participating in multiple mechanisms. It facilitates substance P binding to lymphoblasts, promotes T helper, B cell, and macrophage responses to lymphokines, and facilitates antibody-dependent cytolysis and immune cell adherence. Besides, Mg serves as a cofactor for C'3 convertase and immunoglobulin synthesis. It additionally boasts a significant anti-cancer effect. Chronic Mg deficiency leads to enhanced baseline inflammation associated with oxidative stress, related to various age-associated morbidities. A deficiency of Mg in rodents has been observed to impact the cell-mediated immunity and synthesis of IgG adversely. This deficiency can lead to various complications, such as lymphoma, histaminosis, hypereosinophilia, increased levels of IgE, and atrophy of the thymus. The immunological consequences of Mg deficiency in humans can be influenced by the genetic regulation of Mg levels in blood cells. Mg can also mediate cell cycle progression. There has been a renewed interest in the physiology and therapeutic efficacy of Mg. However, the in-depth mechanisms, their clinical significance, and their importance in malignancies and inflammatory disorders still need to be clarified.

**Conclusions:**

Mg is essential for optimal immune function and regulating inflammation. Deficiency in Mg can lead to temporary or long-term immune dysfunction. A balanced diet usually provides sufficient Mg, but supplementation may be necessary in some cases. Excessive supplementation can have negative impacts on immune function and should be avoided. This review provides an update on the importance of Mg in an immune response against cancer cells and infectious agents and how it regulates inflammation, oxidative stress, cell progression, differentiation, and apoptosis.

## Introduction

Magnesium (Mg) is the second-most abundant cation inside the body's cells, after potassium, and the fourth-most abundant element in the human body (Ca^2+^ > K^+^ > Na^+^ > Mg^2+^)". At birth, the human body possesses an initial Mg content of 760 mg, which subsequently undergoes an increase to approximately 5 g at the age of 4–5 months. The total amount of Mg^2+^ in the body exhibits variation ranging from 20 to 28 g. The majority of Mg^2+^ in the human body, exceeding 99% of the total amount, is found within the intracellular compartment. Its primary storage site is the skeletal system/bones, accounting for approximately 50–65% of the total body Mg^2+^. In conjunction with calcium and phosphorus, Mg^2+^ contributes to the structural composition of the skeleton. Additionally, Mg^2+^ is distributed among muscle tissue, soft tissues, and organs, constituting approximately 34–39% of the total body Mg^2+^. Conversely, a small fraction of Mg^2+^, less than 1–2%, is present in the bloodstream and extracellular fluids [1]. Mg serves as a crucial cofactor in a wide range of biological processes, encompassing over 600 activities. These include the regulation of "cell cycle progression, differentiation, and apoptosis". Additionally, it plays a structural role in nucleic acids through its ability to form complexes with negatively charged compounds like phosphates [2]. Mg plays a role in various biochemical processes, including oxidative phosphorylation, energy generation, protein and nucleic acid synthesis, and glycolysis [3, 4]. This fundamental ion also impacts the excitability of neurons, the reduction of muscle function, and the maintenance of regular heartbeats through its active transport of other ions across cell membranes [5]. According to a recent study conducted by researchers at Basel University, it has been found that immune T cells, which play a crucial role in combating cancer cells and infectious agents, necessitate an adequate amount of Mg in order to effectively detect, activate a response against, and eliminate pathogens [6]. Certain populations, such as athletes and the elderly, may experience a compromised immune system in specific circumstances, particularly in the presence of Mg deficiency [7, 8].

The identification of the quantity of ions present in the microenvironment has been recognized as a crucial factor in the modulation of immune responses. Mg is known to have a significant impact on the immunological response in both the innate and adaptive immune systems [9]. Immunoglobulin production is facilitated by a crucial cofactor [10]. Mg is also essential for the synthesis and distribution of vitamin D, which plays a crucial role in the immune response against viral pathogens [11]. The regulation and transportation of free Mg within the cytosol of immune cells is facilitated by the solute carrier family, which includes the Mg transporter 1 (MAGT1) [12]. The narrative surrounding the interaction between Mg and the immune system emerged during the latter half of the twentieth century [13]. In light of the profound consequences of the ongoing coronavirus pandemic, specifically COVID-19, the substantial worldwide impact of cancer, and the significant role of Mg in enhancing immune health against pathogens and cancers, there has been a resurgence of interest in investigating the importance of maintaining optimal Mg homeostasis for the purpose of enhancing immune health. Mg plays a pivotal role in the immune system through its regulation of the acute-phase response and macrophage function. Numerous studies have provided evidence, indicating that the administration of Mg supplements can effectively decrease the production of cytokines in monocytes after they have been stimulated by toll-like receptors (TLRs). This reduction in cytokine production is achieved by elevating the levels of IĸBα, which subsequently results in the inhibition of nuclear factor kappa-light-chain-enhancer of activated B cell (NF-κB) translocation.

In a mammalian cell, numerous enzymes rely on Mg^2+^ as a necessary cofactor. Additionally, Mg^2+^ plays a critical role in preserving the active structure of macromolecules such as "DNA, RNA, and ATP". It is also involved in regulating second messengers derived from lipids and phosphoinositides, compensating for charge imbalances, and modulating various transporters and ion channels. Additionally, the presence of Mg^2+^ plays a crucial role in regulating the levels of "intracellular free Ca^2+^ and intracellular pH". These factors are significant determinants in various cellular processes such as "cell contraction, secretion, motility, and proliferation". Research conducted on the relationship between cell proliferation and Mg^2+^ has revealed that a deficiency in Mg^2+^ hinders the advancement of the cell cycle, potentially serving as a pivotal factor in "regulating protein translation and cell proliferation". The role of Mg^2+^ in the development or worsening of various pathologies, such as "asthma, diabetes mellitus, hyperlipidemia, atherosclerosis, and hypertension", has been well documented in clinical studies. The inadequacy of Mg^2+^ has been found to be linked to various health conditions, including "epilepsy, migraines, muscular dysfunction, and bone loss". It has been observed that a significant proportion of critically ill individuals, up to 60%, experience a certain level of Mg^2+^ deficiency. This deficiency is often a result of renal losses, which can be attributed to medication usage. Consequently, these patients are at a heightened risk of experiencing severe and potentially life-threatening consequences.

In the context of the immune system, initial observations indicated a clear association between a deficiency in magnesium ions (Mg^2+^) and an escalation in systemic inflammation. This was determined by the presence of heightened levels of "tumor necrosis factor (TNF)-α and other proinflammatory cytokines" in the bloodstream, as well as decreased concentrations of anti-inflammatory cytokines. A deficiency in Mg^2+^ has been found to result in various physiological stress reactions that are pertinent to the immune response. These reactions include endothelial dysfunction and the development of an inflammatory syndrome, which are accompanied by the activation of leukocytes and macrophages. Furthermore, there is an increase in the levels of "proinflammatory cytokines, acute-phase proteins, and free radicals". Additionally, Mg deficiency appears to affect the function of mast cells and their ability to secrete histamine. This Review study was therefore conducted to explore the updates on the importance of Mg in improving the immune system against pathogens, especially cancer and infectious agents.

## Methods

In order to find eligible studies for this review, we conducted a computerized search of MEDLINE, PubMed, EMBASE, and Web of Science databases for all available publications up to December 2022. Our search used keywords such as “Magnesium,” “infectious disease,” “cancer,” “inflammation,” “immune regulation,” and their equivalent terms. Additionally, we reviewed the reference lists of relevant articles and reviews to identify any studies not indexed in these databases. We carefully examined all abstracts and full-text articles and selected the relevant ones for screening and inclusion in this review. Our search was limited to literature in the English language.

## Main text

### Magnesium: sources, absorption, and metabolism

Mg is a vital dietary component for sustaining the physiological functions of living organisms, necessitating regular consumption to meet the recommended intake and mitigate the risk of deficiency. Therefore, it is crucial to not only ascertain the potential origins of Mg but also evaluate its bioavailability and the factors that may impact its absorption and excretion. Mg is ubiquitously present in a variety of food sources, albeit its concentration is subject to diverse factors such as soil and water composition, fertilization practices, preservation techniques, as well as refining, processing, and culinary procedures. Typically, sources of Mg that are regarded as beneficial include “seeds, legumes, nuts (such as almonds, cashews, Brazil nuts, and peanuts), whole grain bread and cereals (such as brown rice and millet), select fruits, and cocoa”. However, it is commonly observed that soil with acidic, light, and sandy characteristics tends to exhibit a deficiency in Mg content. Additionally, the implementation of agricultural practices involving the application of fertilizers with high concentrations of potassium and ammonium has been found to contribute to the depletion of Mg in food [14].

According to the hypothesis, green leafy vegetables are often considered to be a significant dietary source of Mg due to the presence of chlorophyll-bound Mg. Leafy green vegetables, such as lettuce and spinach, typically contain chlorophyll-bound Mg in the range of 2.5–10.5% of the total Mg content. In contrast, other commonly consumed green vegetables, pulses, and fruits contain less than 1% of chlorophyll-bound Mg [15].

The average adult human body typically harbors approximately 1,000 millimoles (mmol) of Mg, which corresponds to a mass range of 22–26 g. Approximately 60% of the total Mg content is found within the skeletal system, with 30% of this fraction being exchangeable. This exchangeable portion serves as a reservoir, playing a crucial role in maintaining the stability of Mg concentration in the serum. Approximately 20% of the total amount is located within skeletal muscle, while another 19% is distributed among various soft tissues. The remaining fraction, less than 1%, is present within the extracellular fluid. The concentration of intracellular Mg is typically tightly regulated, with minimal deviations observed except in exceptional circumstances like hypoxia or prolonged Mg deficiency. Limited knowledge exists regarding the mechanisms implicated in the regulation of intracellular Mg [16].

The current recommended daily allowance (RDA) for Mg in adults is 4.5 mg per Kg of body weight in a day, which represents a decrease from the previous recommendation range of 6–10 mg per kg of body weight per day. The daily nutritional needs are elevated during pregnancy, lactation, and in the aftermath of a debilitating illness. Recent dietary surveys indicate that the average dietary intake in numerous Western countries falls below the recommended daily allowance (RDA) [17].

The intake of Mg is contingent upon the concentration of Mg present in drinking water, as well as the composition of food. "Green leafy vegetables, such as those abundant in Mg-containing chlorophyll, as well as cereal, grain, nuts, and legumes, are all sources of Mg. Chocolates, vegetables, fruits, meats, and fish" exhibit moderate levels of Mg content, while dairy products demonstrate a relatively low-Mg content. The consumption of water can serve as a significant means of obtaining Mg, particularly in the case of “hard water” that may contain Mg levels of up to 30 mg/L. Typically, the consumption of Mg exhibits a direct correlation with energy intake unless a significant proportion of the energy is derived from refined sugars or alcohol. The Mg content of food can be significantly reduced by approximately 85% as a result of the refining or processing process. Moreover, the process of cooking, particularly the act of boiling foods that are rich in Mg, leads to a substantial reduction in Mg content. The potential correlation between the processing and cooking methods employed in food preparation and the observed high incidence of inadequate Mg consumption in numerous populations could be elucidated [18].

The mean Mg consumption of a typical adult is approximately 12 millimoles/day. Furthermore, an estimated 2 millimoles/day of Mg is excreted into the intestinal tract through the secretion of bile, pancreatic juices, and intestinal juices. Approximately 30% of the 6 mmol present in this pool is absorbed, resulting in a net absorption rate of 4 mmol/day. The fractional absorption of Mg in the intestines exhibits an inverse relationship with intake, with a value of 65% observed at low intake and a value of 11% observed at high intake. The majority of absorption takes place within the ileum and colon. In typical consumption patterns, the process of absorption is predominantly passive. A saturable component of Mg absorption can be observed during periods of low-Mg intake. Existing research indicates that parathyroid hormone (PTH) may play a significant role in the regulation of Mg absorption. Phytates present in the dietary intake have the capacity to form complexes with Mg, thereby hindering its absorption. Nevertheless, the quantities of Mg found in a typical diet do not have an impact on the absorption of this mineral. Additional dietary factors that have been hypothesized to impact the absorption of Mg include oxalate, phosphate, proteins, potassium, and zinc [19].

The kidney assumes a significant function in the regulation of Mg levels within the body and the preservation of optimal Mg concentration in the bloodstream. In typical conditions, where approximately 80% of the overall plasma Mg is capable of being filtered, a daily filtration of 84 mmol of Mg occurs. Of this amount, approximately 95% is reabsorbed, resulting in a residual quantity of approximately 3–5 mmol that is excreted in the urine. Roughly 15–20% of Mg that has undergone filtration is reclaimed in the proximal tubular segments, while 65–75% is reabsorbed in the thick ascending limb of Henle (TALH), with the remaining portion being reabsorbed in the distal segments [20].

The transport of Mg in the proximal tubule seems to be predominantly a unidirectional passive process, which relies on the reabsorption of sodium and water, as well as the concentration of Mg in the luminal region. The transport of Mg in the thick ascending limb of the loop of Henle (TALH) is directly correlated with the reabsorption of sodium chloride and the presence of a positive luminal voltage within the segment. In the context of the Tubular Active Loop of Henle (TALH), it has been observed that around 25% of the sodium chloride that undergoes filtration is reabsorbed. This reabsorption occurs through two mechanisms: active transcellular transport, specifically the sodium-chloride-potassium transport, and passive paracellular diffusion. This phenomenon results in the establishment of a beneficial luminal positive potential at the thick ascending limb of the loop of Henle (TALH), which serves as the primary site for Mg reabsorption. The reabsorption of Mg is inversely correlated with the rate of fluid flow in the tubular lumen. The process of reabsorption occurring in the distal convoluted tubule is characterized by active and transcellular mechanisms [21].

### The importance of magnesium in the immune system

Previous studies found that a low-Mg diet can increase the chance of viral infections and encourage rapid metastatic cancer cell development [22]. Recent researches have linked the Mg deficiency with the COVID-19-induced inflammation and oxidative stress [23].

Extensive research during the last two decades has revealed the mechanism by which continued oxidative stress can lead to chronic inflammation, which in turn could mediate most chronic diseases, including cancer, diabetes, cardiovascular, neurological, and pulmonary diseases. Oxidative stress can activate a variety of transcription factors, including NF-κB, AP-1, p53, HIF-1α, PPAR-γ, β-catenin/Wnt, and Nrf2. Activation of these transcription factors can lead to the expression of over 500 different genes, including those for growth factors, inflammatory cytokines, chemokines, cell cycle regulatory molecules, and anti-inflammatory molecules. In this way, oxidative stress activates inflammatory pathways leading to the transformation of a normal cell to a tumor cell, tumor cell survival, proliferation, chemoresistance, radioresistance, invasion, angiogenesis, and stem cell survival. Overall, it can be suggested that oxidative stress, chronic inflammation, and cancer are closely linked [24].

**Fig. 1:**
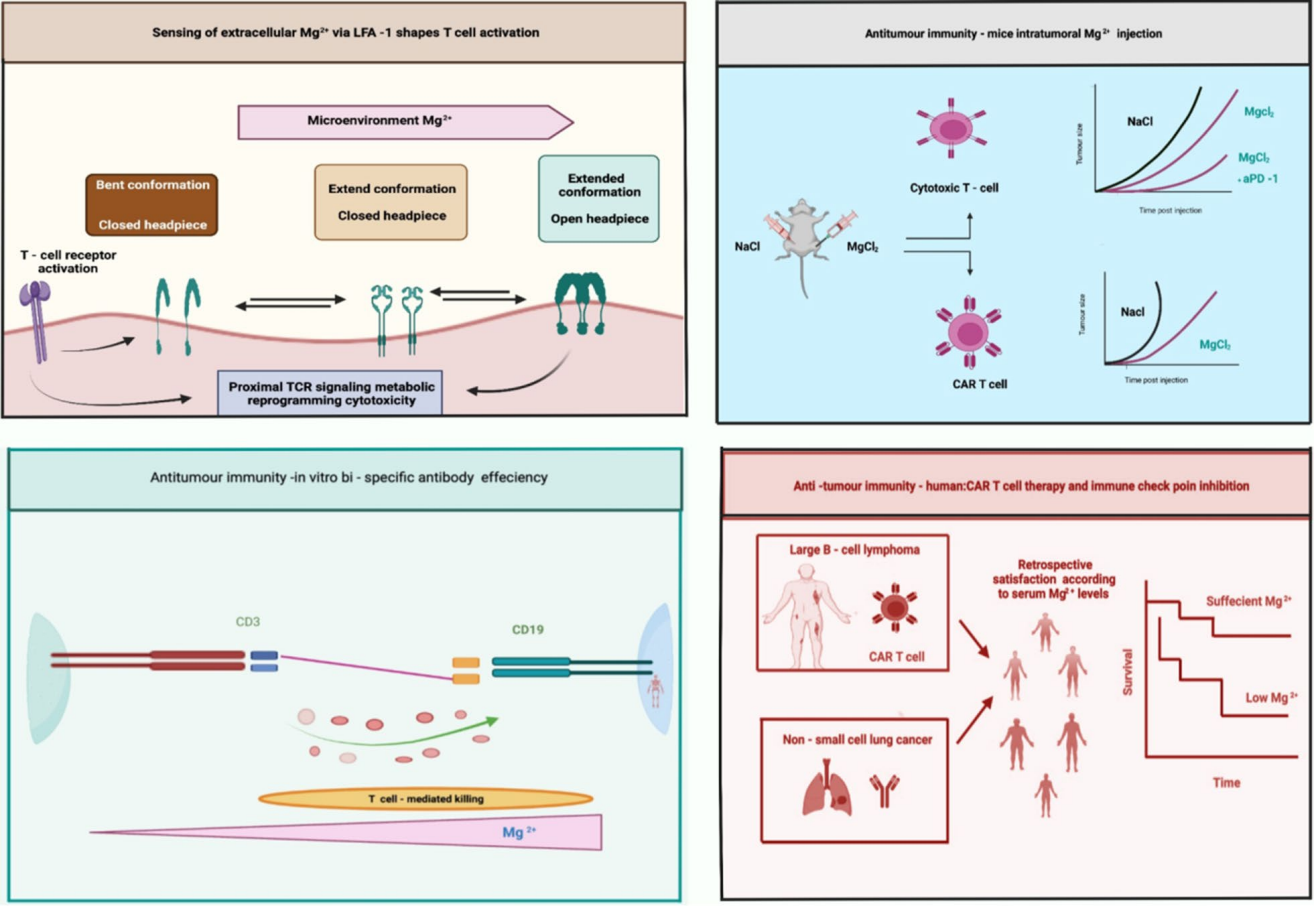
On CD8^+^ T cells, LFA-1 binds extracellular magnesium, improving immunological responses to pathogens (e.g., tumor cells)

The mechanism of Mg in the immune system has been a mystery until today. Recently, Lötscher and colleagues conducted preclinical and clinical research to determine the role of Mg in regulating immune function. They found that Mg is necessary for the proper function of the cell surface protein on CD8^+^ T- lymphocytes termed (LFA-1) (lymphocyte function-associated antigen 1) [6]. LFA-1 is associated with the regulation of leukocyte function and directly includes information on the constitutions of the microenvironment as a determining factor of outside-in signaling function T cell initiation at the synapse and immune cell blocking are involved in T cell transportation from blood vessels into the tissues in the immediate vicinity. This is associated with LFA-1, a transmembrane receptor protein called an integrin that promotes the association between the cell with its extracellular matrix. For target cells, LFA-1 serves as a docking site in T cell initiation strategies [25]. In the clinical phase, Lötscher et al. found that low serum Mg levels were linked with more advanced disease conditions and shorter inclusive survival in chimeric antigen receptor (CAR) T cells and immune checkpoint antibody-treated patients. In individuals treated with CAR T cells or immune checkpoint antibodies, decreased serum Mg levels were linked to speeding up disease onset and shorter overall survival. As a result, LFA-1 uses information about the microenvironment as a direct indicator of the outside-in signaling route [6]. This review establishes a strong relationship between nutritional sensing as well as co-stimulation, pointing toward the axis of Mg with LFA-1 as a potential therapeutic biological network. In other words, the CD8^+^ T cell effector response is catalyzed by Mg. Mg sensing via the co-stimulatory protein LFA-1 is becoming increasingly important in the interplay of cancer response and infections (Fig. 1). In cancer immunotherapy, where cytotoxic T lymphocytes are employed to destroy cancer cells, the response of Mg is crucial for T cell response. The capacity of T lymphocytes for antigen-directed cytotoxicity has been widely accepted for engaging the immune system in the management of cancer. Current findings reveal the molecular and cellular biology of the T cells that links to new approaches in this mechanism, including checkpoint inhibition, adoptive cellular treatment, and cancer vaccinology. The gene expression nature of low-and high-Mg cells resulted in the modification of various genes, some regulating cell progression. Wolf et al. demonstrated this effect in mammary epithelial HC11 cells. They showed that a low-Mg diet can lead to G_0_/G_1_ arrest and overexpression of glutathione S-transferase [26].

**Fig. 2:**
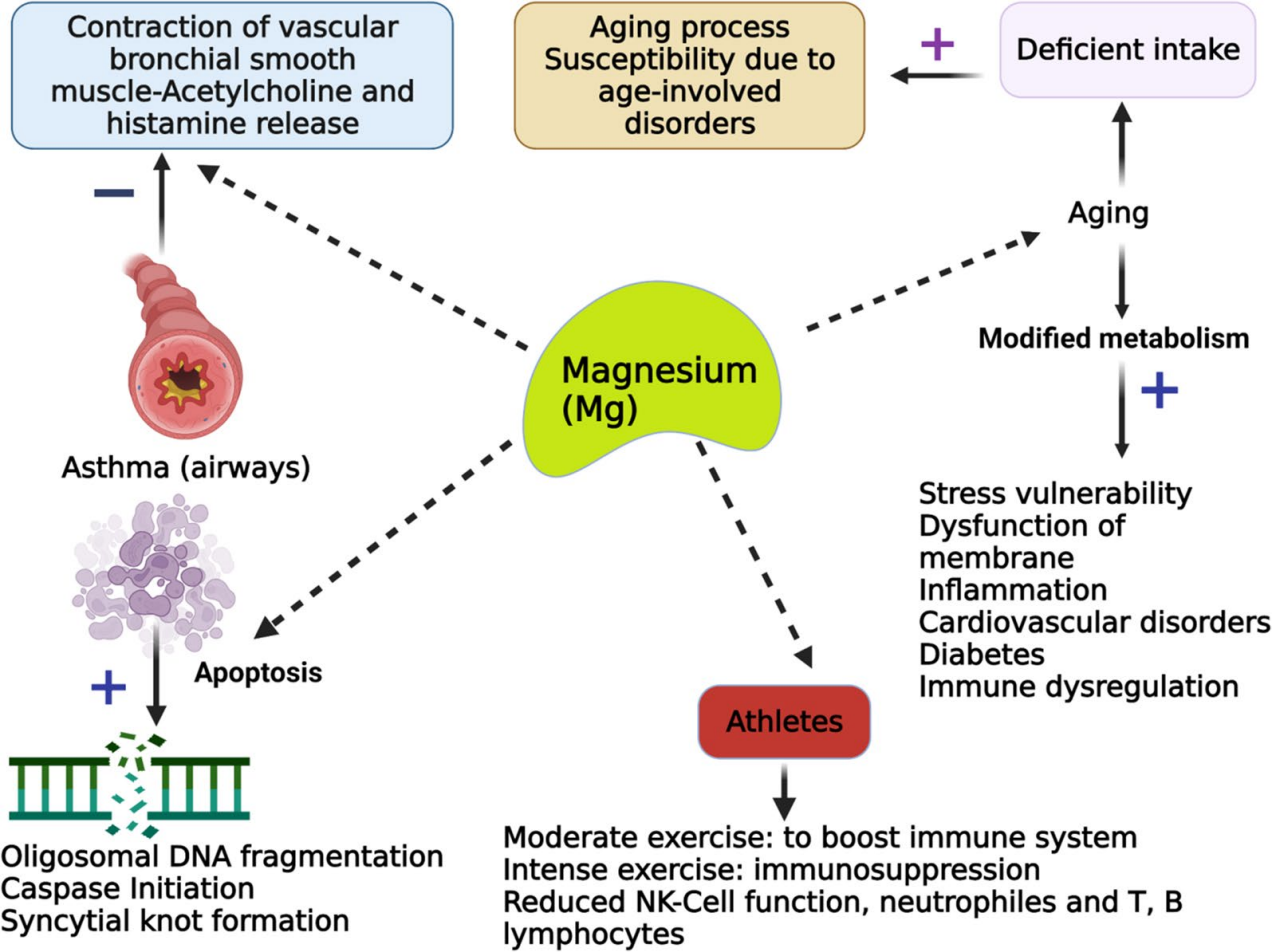
Association between Mg with a few characteristics linked with the human immune system

Mg is required for the immune system's basic cellular and bodily homeostasis processes. It regulates the development, balance, and activation of immune responses in both innate and acquired immune systems. Mg deficiency, which is common in old age, is strongly linked to inflammation through a variety of mechanisms [27]. The proinflammatory effects of Mg deficiency are mainly mediated by N-methyl-d-aspartate (NMDA) receptor, and NF-κB, which can result in oxidative stress in severe cases [28]. The recognition of X-associated Mg-deficit immunodeficiency (XMEN), a genetic condition associated with severe chronic Epstein–Barr viral (EBV) contamination and EBV-induced neoplasia, validates the crucial role of Mg in regulating immune system [29, 30]. The proper activation of inositol triphosphate (IP3) progression, PLC-g1, and protein kinase Cu phosphorylation, including calcium mobilization through store-modulated calcium introduction, requires the MAGT1-dependent Mg flow. In T cells and B cells, MAGT1 deficiency lowered cytosolic free Mg and hindered Mg absorption. Mg supplementation has been shown to improve bronchodilation including the function of the lungs in asthma patients [31]. Mg is an important cofactor for the synthesis of immunoglobulin (Ig), C3 convertase, adhesion of immune cells; antibody-based cytolysis, IgM lymphocyte binding, macrophage response to lymphokines, and T helper–B cell adherence [32]. All experiments were conducted on experimental subjects with an Mg-deficient diet, and as a result, the animals' polymorphonuclear cell quantity and response, as well as the number of neutrophils, were altered, resulting in improved phagocytosis [27]. It has been demonstrated that an insufficient amount of Mg in experimental subjects elevated inflammation, amplified immune stress functions, and reduced specific immune reactions [9]. (Figure 2)

#### Inflammation

**Fig. 3:**
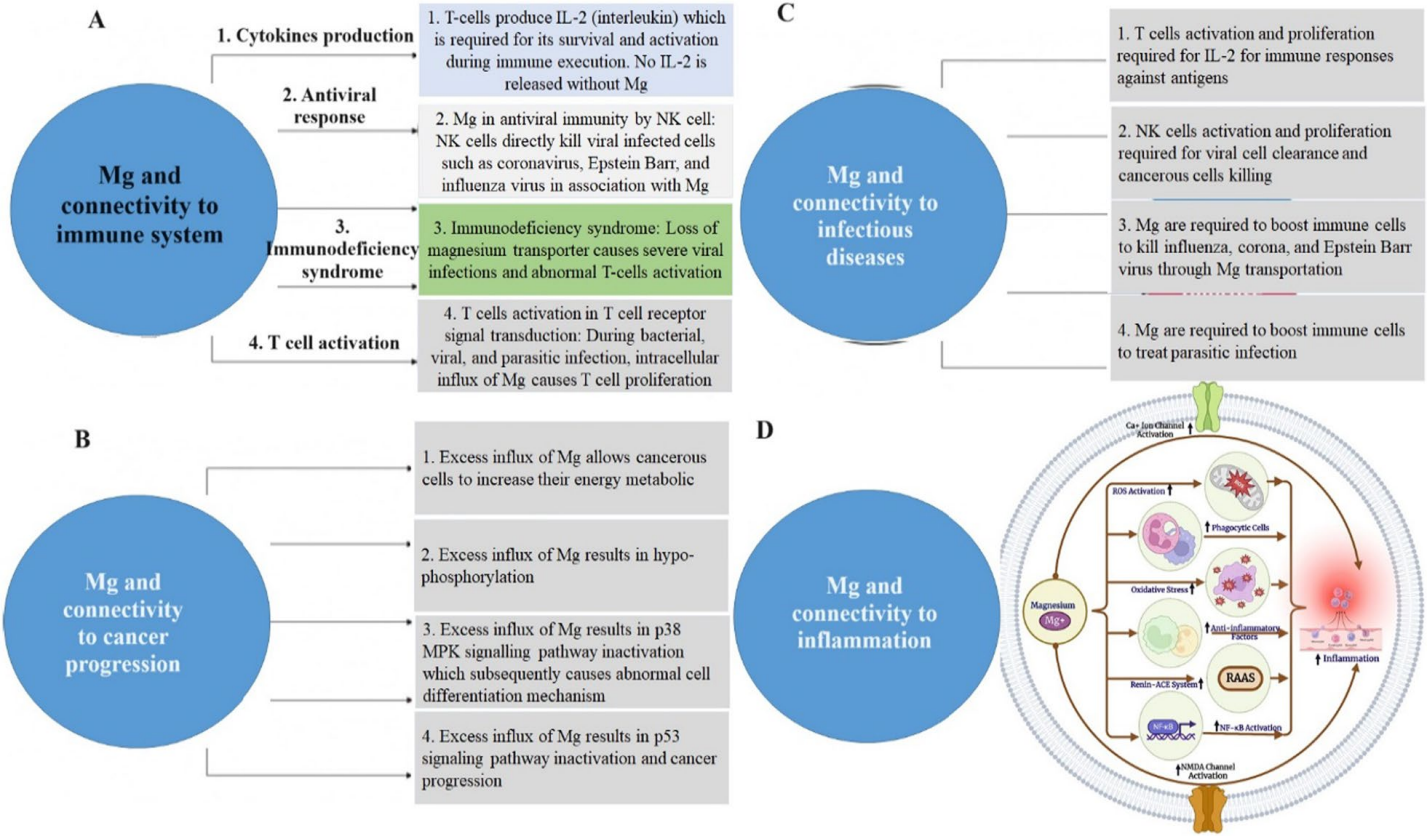
Role of magnesium in various physiological and pathological consequences. Mg is associated with immune response (**A**), cancer progression (**B**), infectious diseases (**C**), and inflammation (**D**). Inflammation is induced by magnesium depletion via numerous signaling mechanisms [40, 103]. NMDA indicates N-methyl-D-aspartate; RAAS, the renin–angiotensin–aldosterone system

Studies conducted in vitro have found that chronic inflammation related to Mg deficiency may be linked to the production and release of interleukin-1 (IL-1), tumor necrosis factor (TNF), as well as the activation of phagocytosis, calcium channel opening, NMDA receptor activation, NF-B signaling, and stimulation of nitric oxide with inflammatory markers [33]. Mg is an essential mineral that is involved in numerous physiological processes, including vascular and inflammatory functions. Studies have shown that Mg deficiency can promote platelet agglomeration, which can affect micro vascular functions, and also limit the growth and migration of endothelial cells. Additionally, research suggests that the stimulation of the IL-33/ST2 axis, a key pathway in inflammation, can lead to decreased Mg levels in severely inflamed tissues, highlighting the importance of Mg in the inflammatory pathway [34]. Endothelial dysfunction, which has been linked to inadequate Mg levels, can also trigger the release of inflammatory mediators [35]. MgSO_4_, also known as magnesium sulfate, has been shown to have anti-inflammatory effects by preventing the overproduction of inflammatory mediators such as NF-κB through the activation of phosphoinositide 3-kinase. This compound has also been found to block L type ion channels in inactive macrophages in mice [36]. In humans, studies have linked low serum Mg levels and inadequate dietary intake of Mg with systemic inflammation [37]. Less Mg consumption is positively associated with higher levels of systemic inflammation and metabolic syndrome in individuals who consume insufficient Mg [38, 39]. (Figure 3)

#### Oxidative stress

**Fig. 4:**
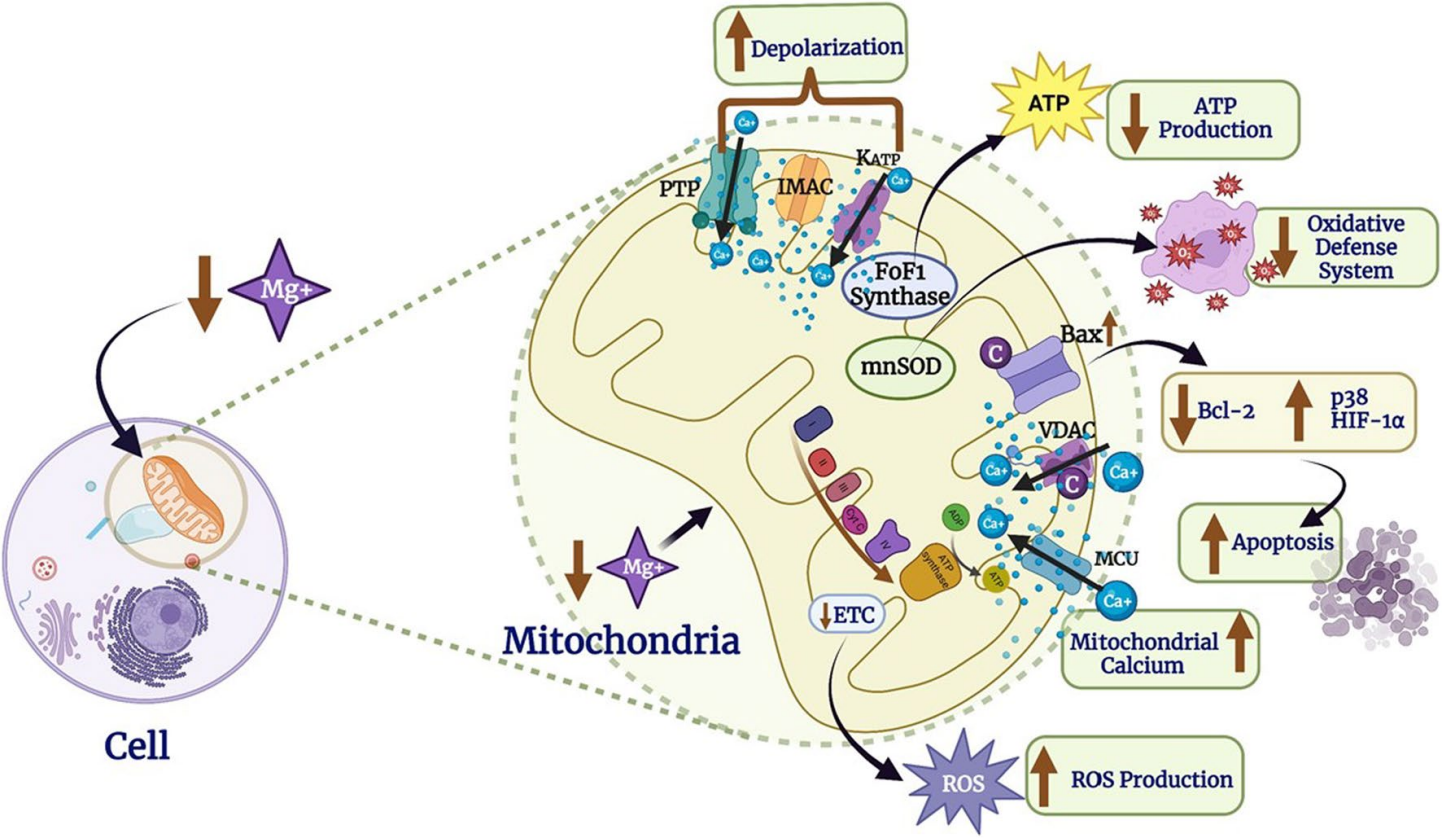
Intracellular Mg deprivation induces oxidative stress including malfunction in mitochondria [40]. ATP indicates adenosine triphosphate; ETC, electron transport chain; F0F1-ATPases, membrane-bound ATP synthases; IMAC, inner membrane anion channel; KATP, ATP-sensitive K channel; MnSOD, manganese superoxide dismutase; MRS2, mitochondrial RNA splicing 2; PTP, permeability transition pore; ROS, reactive oxygen species; VDAC, voltage-dependent anion channel

Elevated oxidative stress and impaired antioxidant defense barriers have been linked to Mg deficiency. Mg deficiency is associated with enhanced generation of free oxygen radicals in several tissues. Tissue damage is caused by free radical formation because of increased generation of superoxide anion by inflammatory cells [40]. Antioxidant enzymes promotes oxygen peroxide generation, and reduces cellular, tissue antioxidant concentrations [41, 42]. Mg deficiency is observed in various animal models for improving lipid peroxidation mechanisms while lowering of hepatic glutathione, vitamin-E levels, and superoxide dismutase, which further leads to an increase in oxidative stress [43]. Mg shows antioxidant activities that scavenge free oxygen radicals, potentially by activating mitochondrial antioxidants. Low serum Mg concentrations have been reported to influence Mg conveyor TRPM7 and SLC41A cells. Diabetic mice with Mg deficiency were found to have elevated mitochondrial oxidative stress, which causes cardiac diastolic malfunction that can be prevented by Mg supplementation. These data suggest the role of Mg as a mitochondrial antioxidant [44]. Mg deficiency in several experimental studies reported altered mitochondrial functions such as alteration of respiration, increased mitochondrial ROS generation, and blockade of antioxidant defense system (e.g., superoxide dismutase, vitamin E, catalase, glutathione) [45]. Calcium is induced by the mitochondrial calcium uniporter [46], pro-survival signaling is reduced [47], stimulating activation of the mitochondrial potassium channel which is ATP-sensitive to the anion channel in the inner membrane [48]. According to a study, Mg supplementation improves mitochondrial function through various mechanisms like mitochondrial ROS inhibition, modulation of permeability, and mitochondrial transition pore opening [49]. “Inflammaging” refers to the chronic, low-grade inflammation that refers to aging which is associated with various tissues and organs (gut microbiota), and is evaluated by a complex balance between pro- and anti-inflammatory responses. The main source of inflammatory stimuli involves endogenous, misplaced, or modulated components resulting from impaired or dead cells and organelles decorated by receptors of the innate immune system. Age-associated mitochondrial dysfunction is linked to inflammation (source of oxidative stress) and “self-garbage” (mtDNA, cardiolipin, or formyl peptides) that may be detected by macrophages [50] (Figure 4). Mg insufficiency disrupts the electron transport chain and facilitates the generation of reactive oxygen species. The reduced protein expression of manganese superoxide dismutase, including catalase, is indeed driven by Mg deprivation, affecting the antioxidant defensive reaction. Mg deprivation reduces ATP biosynthesis via down-regulating ATP synthase (F_0_F_1_). Intracellular Mg insufficiency prohibits Mg from accessing mitochondria via the mitochondrial RNA splicing 2 (MRS2) protein and triggers Mg efflux via SLC41A3. Mg deprivation enhances apoptosis and involves elevating cytochrome C discharge via Bax or the voltage-dependent anion channel (VDAC), suppressing anti-apoptotic proteins just like Bcl-2 family, with promoting pro-apoptotic proteins like HIF-1, including p38/JNK. Mg scarcity triggers depolarization of the mitochondrial membrane (m) via enhancing the permeability of the mitochondrial permeability transition pore (PTP), ATP-sensitive K channel (KATP), and inner membrane anion channel (IMAC). Mg deficit elevates mitochondrial Ca^2+^ (Ca m) through the mitochondrial Ca^2+^ uniporter (MCU). Mg deficit promotes Ca leakage from mitochondria via VDAC.

### Magnesium with cancer: an emphasis on animal models

Various studies conducted in animal models have shown that Mg may have a protective effect against certain types of cancer in the early stages of chemical carcinogenesis [51, 52]. For example, it has been reported to inhibit lead (Pb) and nickel (Ni)-related lung cancers in mice and to inhibit nickel-induced carcinogenesis in rat kidneys [53]. Mg has also been shown to protect rats from fibro-sarcomas caused by 3-methyl-cholantrene and to reduce c-myc expression and ornithine decarboxylase activity in the intestinal mucous membrane [54, 55]. As a result, it is suggested that Mg could potentially be used as a useful chemotherapeutic agent for treatment. One study found that Mg in the diet slowed tumor development in young male rats with Walker 256/M1 carcinosarcomas by inhibiting glutathione synthesis, which requires Mg as a cofactor [56]. Diet that includes Mg can inhibit the growth of certain types of cancer cells, including Lewis lung carcinoma, mammary adenocarcinoma, and colon cancer [57]. Inflammation is known to be linked to the development of cancer, and in the early stages of cancer, inflammatory mediators can promote invasion and metastasis [58]. Mg deficiency can lead to the activation of TNF, as well as IL-1 and IL-6 [59], which can increase the potential for cancer cells to spread [60]. This suggests that Mg is necessary for the proper functioning of the NM23-H1 gene product, which suppresses metastasis. Inadequate Mg levels, or hypomagnesemia, may contribute to more advanced cancer symptoms and increased metastasis [61, 62].

### Low magnesium with cancer: human studies

Mg is an essential mineral that plays a crucial role in various biological processes in the human body, including DNA synthesis, protein synthesis, and energy metabolism. Mg deficiency has been linked to several health problems, including cancer. In cancer patients, Mg deficiency can impact treatment outcomes and overall quality of life. Research suggests that low levels of Mg can contribute to an increased risk of developing certain types of cancer, such as colorectal cancer, pancreatic cancer, and breast cancer. Mg also plays a vital role in regulating inflammation in the body. Inflammation is a significant contributor to the development of cancer and can worsen cancer-related symptoms. Therefore, Mg deficiency can exacerbate inflammation, potentially leading to more severe cancer-related complications. Several epidemiological studies have found a potential association between dietary Mg intake and various types of cancer. For instance, a high amount of Mg in drinking water may provide protection against liver and esophageal cancers [63, 64]. Furthermore, a recent meta-analysis showed that a linear relationship exists between higher dietary Mg intake and reduced cancer mortality, with a 5% decrease in cancer mortality observed for every 100 mg/d increase in Mg intake [65]. In addition, some studies have found that Mg deficiency may be linked to an increased risk of colon cancer [66, 67], while others have observed a significant inverse association between dietary Mg and colon cancer in men, but not women [68]. Interestingly, lower dietary Mg intake may be associated with increased production of N-nitroso compounds, which are carcinogens, in colon cancer patients [69]. A genetic variant of TRPM7 has been found to be associated with adenomatous and hyperplastic polyps that can lead to colon cancer, highlighting a potential link between Mg and colon neoplasia. Mg uptake and homeostasis are regulated by a ubiquitous ion channel. While the role of Mg in lung cancer is still a matter of debate, it is necessary for maintaining genomic stability [70]. However, there is a lack of data on the relationship between dietary Mg intake and lung cancer. Mahabir et al. concluded that low dietary Mg intake was associated with poorer DNA repair capacity and an increased risk of developing lung cancer. The passage discusses a possible link between dietary Mg intake and lung cancer risk, as well as the role of inflammation in cancer development in people with Mg deficiency [71]. The findings suggest that having a diet rich in Mg may decrease the risk of lung cancer. However, when low-Mg intake and suboptimal dose–response relationship curve are present together, the odds ratio for lung cancer increases to 2.36, regardless of gender. The risk appears to be higher among older populations, heavy smokers, drinkers, and people with a family history of cancer, those with small cell lung cancer, and those with late-stage disease [71]. These results need to be confirmed in prospective studies. Furthermore, people with solid tumors often have low serum Mg concentrations, even after treatment, and regardless of cancer stage [72]. The reduced Mg uptake may lead to increased inflammation, which is significant in cancer development in Mg-deficient individuals [73].

### Magnesium interactions with anti-cancer agents

#### Ascorbic acid

L-ascorbic acid (AA), also termed vitamin C, is a polyunsaturated fatty acid with antioxidant and prooxidant properties. The impact of AA on cancer cells is based on the *hormetic* effect, characterized by low-dose stimulation and high-dose inhibition. In other words, AA is only effective against cancer cells in higher quantities because of its prooxidant properties. The cellular absorption of AA is determined by the sodium-dependent vitamin-C transporter family-2 (SVCT2). Low SVCT2 expression on tumor cells is tumoricidal at high doses of AA but has a proliferative effect at low doses of AA. In contrast, tumor cells with high SVCT2 expression exhibit anti-cancer outcomes even at low AA concentrations [74]. Cho et al. demonstrated that Mg ions can enhance the expression of SVCT 2, which increases its V_max_ value. Molecular analysis data have confirmed the enhanced expression of cancer proliferation markers in the hormetic dose response [75]. These findings addressed that Mg can enhance the anti-cancer effects of AA.

#### Valproic acid

Bladder cancer cell proliferation was shown to be reduced after elevated MgCl_2_ or MgSO_4_ therapy. Li et al. demonstrated that MgCl_2_ can activate the G_0_/G_1_ cell cycle arrest, autophagy, apoptosis, and endoplasmic reticulum of bladder cancer cells but not their migratory potential. In addition, a fraction of CD44 or CD133 positive cells did not differ significantly between MgCl_2_ treated and control cells. Next, Li et al. added valproic acid (VPA) to enhance the therapeutic effect of Mg. *In vivo*, MgCl_2_ and VPA fusion inhibited UC3 cell proliferation, migration, and tumorigenicity, as expected. Furthermore, Wnt signaling (which regulates the progenitor cell homeostasis, thereby controlling hematopoiesis) was inhibited, while ERK signaling was increased with the combination treatment in treated cells [76]. This study demonstrated that Mg and VPA have synergistic effects on bladder cancer cells.

#### Magnesium chloride (MgCl_2_)

The effects of MgCl_2_ on cell migration, apoptosis, expression of EMT (epithelial-to-mesenchymal transition) markers including expression of V-H + -ATPase, myosin II (NMII), and the transcription factor NF-kB were studied. Santos et al. found that MgCl_2_ causes apoptosis and significantly slows migration in cancer cells with varying metastatic potentials. MgCl_2_ inhibits invasion and metastasis by lowering V-H plus ATPase with myosin II expression, suppressing vimentin expression, and increasing E-cadherin expression, implying function for MgCl_2_ in EMT reversing. In addition, MgCl_2_ inhibits NF-kB expression while promoting chromatin condensation. MgCl_2_ appears to have a propitious prophylactic with remedial aspects in endocrine cancer based on these studies [77].

### Infectious diseases in old age

Infections are a leading source of illness and mortality in the elderly, resulting in organ modification, functional reduction, poly-morbidity, debility, affliction, including associated medical interventions, due to various physiological alterations with progressive worsening of homeostatic mechanisms [78], as well as changes in age-related immunological response [79]. According to evidence from infectious disease hospitalizations in the USA, from nationwide inpatient data between 1998 to 2006, the death rate from acute contamination was more than fifty-fold higher in those above 65 than in people in their 30s and 50s [80]. As people get older, their immune systems lose their natural ability to fight infections, increasing their risk of infection, neoplasms, and autoimmune diseases, as well as their ability to heal skin wounds [81]. Illness load is linked to functional deterioration and a decrease in immune system competence rather than chronological age. Older persons with chronic conditions are more receptive to common contamination and are involved in lower immunization reactions as compared to those without chronic problems (e.g., failure of heart, COPD, diabetes). Table 1 describes various inflammation markers and the role of Mg [82–90]. Mg is an anti-inflammatory mineral with innate immune system functions, and the relaxing of bronchial smooth muscle in COVID-19 calls for more research. Nouri-Majd et al. (2022) conducted a cross-sectional study with 250 COVID-19 patients between the ages of 18 and 65. To quantify dietary Mg consumption, a validated 168-item online food frequency questionnaire (FFQ) was used. The severity of COVID-19 was determined using the COVID-19 Treatment Guidelines, and symptoms were assessed using a common questionnaire. Participants had a 44.1 mean age and 46% of them had severe COVID-19. The serum levels of inflammatory biomarkers, such as CRP, were lower in patients in the highest tertile of dietary Mg consumption than in those in the lowest tertile. They found that a decreased risk of severe COVID-19 was connected with higher dietary Mg consumption. The important evidence for how Mg may lessen COVID-19 symptoms and inflammation is its role in reducing asthma (lung inflammation) symptoms, which has been previously described [91, 92]. It is conceivable that Mg may lessen COVID-19 symptoms by lessening lung inflammation [93, 94]. Additionally, IL-6, a proinflammatory cytokine and a potential target for COVID-19 treatments, has been linked to Mg deficiency [95]. In accordance with recent research, Mg may help prevent COVID-19 symptoms. In one retrospective cohort study, blood samples from 306 COVID-19 patients who were admitted to Tongji Hospital in Wuhan, China, were examined for the presence of hazardous and important metals [96]. According to the study's findings, those with more severe COVID-19 symptoms had lower Mg levels. The goal of a retrospective cohort research on 629 COVID-19 hospitalized patients was to determine whether serum Mg levels and cardiac damage and disease prognosis were related [97]. Determining the significance of low-Mg levels in COVID-19 patients was the goal of another retrospective cohort investigation [98]. Eighty-three patients who were receiving medical care at Wuhan Third Hospital's Guanggu Hospital District were examined. The patients' serum Mg levels were examined, and the patients were divided into various groups according to the severity of their diseases (moderate, severe, critical). In their analysis of more than 300 patients, Zeng et al. discovered that although all values were within the standard range, severe cases had significantly lower levels of Mg than mild and moderate cases [96]. Throughout the clinical history since the beginning of the condition, this discrepancy was often observed. Low-Mg levels were also identified as a mortality risk factor in COVID-19 patients. Mg levels were much lower in the 63 deceased people than in the 396 survivors, according to a retrospective study on a total of 459 verified cases [99]. Patients with severe COVID-19 symptoms might need to stay in hospitals with intensive care units (ICU). It is interesting to note that up to 60% of critically sick patients in ICU have been documented to have some degree of Mg deficiency, predisposing these patients to catastrophic, maybe fatal effects due to the ensuing hypokalemia and hypocalcemia [100]. Unfortunately, there is currently no direct data available about the significance of Mg in COVID-19, likely because Mg is not often tested in significant databases and studies [101]. Mg is an essential mineral that plays a crucial role in the human body. One of its important functions is its interaction with vitamin D metabolism, as Mg serves as a cofactor for vitamin D synthesis. A deficiency of Mg can lead to a decrease in vitamin D formation from its precursors. While most of the research on the connection between Mg status and immune function is based on animal experiments [93]. These studies have consistently shown that Mg deficiency can disrupt the inflammatory response and increase the risk of infections. Therefore, maintaining adequate levels of Mg in the body is essential for optimal immune function and overall health [102].

**Table 1:** Relevance of Mg deficiency in various pathological abnormalities as investigated in animal model and clinical trials

Biomarkers	Findings	Ref.
↑IL-1a, IL-6, NO, and VCAM	Mg deficiency promoted inflammation and angiogenesis. Low content of Mg provoked increased level of IL-1a, IL-6, NO, and VCAM	[82]
↑MCP-1, MT, RANTES	Decreased level of Mg in erythrocytes of atherosclerotic patients. Decreased NK cells cytotoxicity potential	[83]
Alkaline phosphatase	Reduction in dietary Mg by 50% resulted in reduced bone mineral content and the volume of distal femur	[84]
Elastin/collagen ratio	Long term Mg deficiency in diet results in cardiovascular risk in rats	[85]
Plasma IL-6, fibrinogen, and erythrocytic lysophosphatidylcholine	Long term Mg deficiency in diet of aging rats is related to high blood pressure, inflammation, and oxidative distress	[85, 86]
CRP, IL-6, TNF-α R2, soluble VCAM-1	Dietary Mg connectivity to inflammatory biomarkers and endothelial dysfunction in post-menopausal women in a cohort study	[87]
IL-8, NF-kB	Potential interplay of NF-kB and PPARY in cultured human endothelial cells	[88]
IL-1, IL-6, and TNF-alpha	Dietary Mg deficiency was induced in rodents to execute inflammatory responses as evidenced with high levels of ILs in circulation	[89]
ROS, ↑ 8-hydroxy-deoxyguanine, and ↑ IL-1 and IL-6	Low Mg was related to aging, oxidative distress, atherosclerosis, and other vascular disorders.	[90]
Markers not reported	Mg sulfate reduced asthma in patients not responding to conventional medicine and steroidal drugs. Similarly, 68% of children hospitalization was reduced by Mg sulfate	[91]
Markers not reported	Intravenous administration of Mg sulfate reduced acute asthmatic inflammation when not responded to the first-line treatment	[92]
IL-6, ↑ alpha2-macroglobulin and alpha1-acid glycoprotein and ↑ fibrinogen	Inflammatory responses in response to acute deficient Mg in rat	[93]
↓ IL-6, CRP, and NF-kB	Clinically, Mg was recommended in covid-19 infected patients.	[95]
Serum Mg level	No positive correlation between Mg deficiency and covid-19 infected myocardial diseases.	[97]
A comprehensive review report	Mg deficiency is linked to diabetes, heart failure, and other cardiac issues	[40]

CRP, C-reactive protein; IL, interleukin; MCP-1, Mg, magnesium; monocyte chemoattractant protein-1; NF-kB, nuclear factor kappa-light-chain-enhancer of activated B cells; NO, nitric oxide; RANTES, regulated upon activation, normal T cell expressed and secreted; ROS, reactive oxygen species; TNF, tumor necrosis factor; VCAM, vascular cell adhesion molecule

## Conclusions

Several published studies have reported that diets low in Mg have been linked to adverse effects on the immune response, oxidative stress, and inflammatory markers in animal models. Despite the current limitations in available evidence, the majority of published data indicates that Mg possesses chemo-preventive properties. This suggests that enhancing Mg intake could potentially serve as a cost-effective and economically viable strategy for immune regulation and preventing cancer. The existing correlations between Mg and tumors in the field of clinical oncology warrant further investigation to enhance our understanding of the role of Mg in tumor development. This review could potentially shed light on the potential benefits of optimizing Mg homeostasis as a therapeutic approach in cancer treatment. Mg is a crucial mineral that plays a vital role in maintaining the optimal functioning of the immune system, as extensively evidenced in scientific literature. The role of Mg in various physiological processes has been extensively explored, including its impact on inflammation, apoptosis, thymocyte gene expression, as well as histological and cytological effects in animal models. Furthermore, investigations have also examined the association between Mg and asthma, the immune system in athletes, aging processes, and apoptosis in humans. While mineral deficiencies are infrequent, certain populations are more susceptible to inadequate mineral intake and should prioritize the consumption of a well-balanced diet to ensure sufficient supply. Supplementation may be necessary to address a Mg deficiency in certain exceptional cases. Nevertheless, it is crucial to acknowledge that an overabundance of mineral supplements can potentially have detrimental impacts on the immune system. Hence, it is imperative that any type of nutrient supplementation is subject to medical approval and adheres to the prescribed dosages. Nevertheless, there remain several inquiries that necessitate the implementation of more comprehensive and multifaceted experimental methodologies. Additional investigation is required to elucidate the precise involvement of Mg in various distinct biological processes that are directly or indirectly associated with the immune system, as its role in these processes remains ambiguous.

## Data Availability

NA.

## Abbreviations

AA: L-ascorbic acid
ATP: Adenosine triphosphate
CAR T cells: Chimeric antigen receptor T cells
COPD: Chronic obstructive pulmonary disease
COVID-19: Coronavirus disease 2019
EBV: Epstein–Barr virus
EMT: Epithelial-to-mesenchymal transition
ERK: Extracellular signal-regulated kinase
FFQ: Food frequency questionnaire
HIF-1: Hypoxia inducible factor 1
ICU: Intensive care unit
Ig: Immunoglobulin
IL: Interleukin
IĸBα: Inhibitor of kappa B alpha
IP3: Inositol triphosphate
KATP: ATP-sensitive K channel
LFA-1: Lymphocyte function-associated antigen 1
MAGT1: Mg transporter 1
MCU: Mitochondrial calcium uniporter
Mg: Magnesium
MgCl_2_: Magnesium chloride
MgSO4: Magnesium sulfate
MRS2: Mitochondrial RNA splicing 2
mtDNA: Mitochondrial DNA
NF-κB: Nuclear factor kappa-light-chain-enhancer of activated B cell
NMDA: N-methyl-d-aspartate
PLCG1: Phospholipase C gamma 1
PTP: Permeability transition pore
ROS: Reactive oxygen species
SVCT2: Sodium-dependent vitamin-C transporter family-2
TLR: Toll-like receptor
TNF: Tumor necrosis factor
TRPM7: Transient receptor potential cation channel subfamily M member 7
VDAC: Voltage-dependent anion channel
VPA: Valproic acid
XMEN: X-associated Mg-deficit immunodeficiency
