# Correction: A narrative review on the role of magnesium in immune regulation, inflammation, infectious diseases, and cancer

**DOI:** 10.1186/s41043-023-00461-8

**Published:** 2023-11-02

**Authors:** Sumel Ashique, Shubneesh Kumar, Afzal Hussain, Neeraj Mishra, Ashish Garg, B. H. Jaswanth Gowda, Arshad Farid, Gaurav Gupta, Kamal Dua, Farzad Taghizadeh‑Hesary

**Affiliations:** 1 Department of Pharmaceutics, Pandaveswar School of Pharmacy, Pandaveswar, West Bengal 713378 India; 2 Department of Pharmaceutics, School of Pharmacy, Bharat Institute of Technology (BIT), Meerut, UP 250103 India; 3 Department of Pharmaceutics, College of Pharmacy, King Saud University, 11451 Riyadh, Saudi Arabia; 4 Department of Pharmaceutics, Amity Institute of Pharmacy, Amity University Madhya Pradesh (AUMP), Gwalior, MP 474005 India; 5 Department of Pharmaceutics, Guru Ramdas Khalsa Institute of Science and Technology (Pharmacy), Jabalpur, Madhya Pradesh India; 6 Department of Pharmaceutics, Yenepoya Pharmacy College & Research Centre, Yenepoya (Deemed to Be University), Mangalore, 575018 India; 7 Gomal Center of Biochemistry and Biotechnology, Gomal University, D.I.Khan, KPK Pakistan; 8 School of Pharmacy, Suresh Gyan Vihar University, Mahal Road, Jagatpura, Jaipur, India; 9 Discipline of Pharmacy, Graduate School of Health, University of Technology Sydney, Ultimo, NSW 2007 Australia; 10 Faculty of Health, Australian Research Centre in Complementary and Integrative Medicine, University of Technology Sydney, Ultimo, Australia; 11 ENT and Head and Neck Research Center and Department, The Five Senses Health Institute, School of Medicine, Iran University of Medical Sciences, Tehran, Iran; 12 Department of Clinical Oncology, Iran University of Medical Sciences, Tehran, Iran

**Correction: Journal of Health, Population and Nutrition (2023) 42:74** **https://doi.org/10.1186/s41043-023-00423-0**

Following publication of the original article [1], Figs. 3 and 4 and Table 1 were found to contain overlap with a separate article [2]. The corrected figures and table are shown below.

**Fig. 3:**
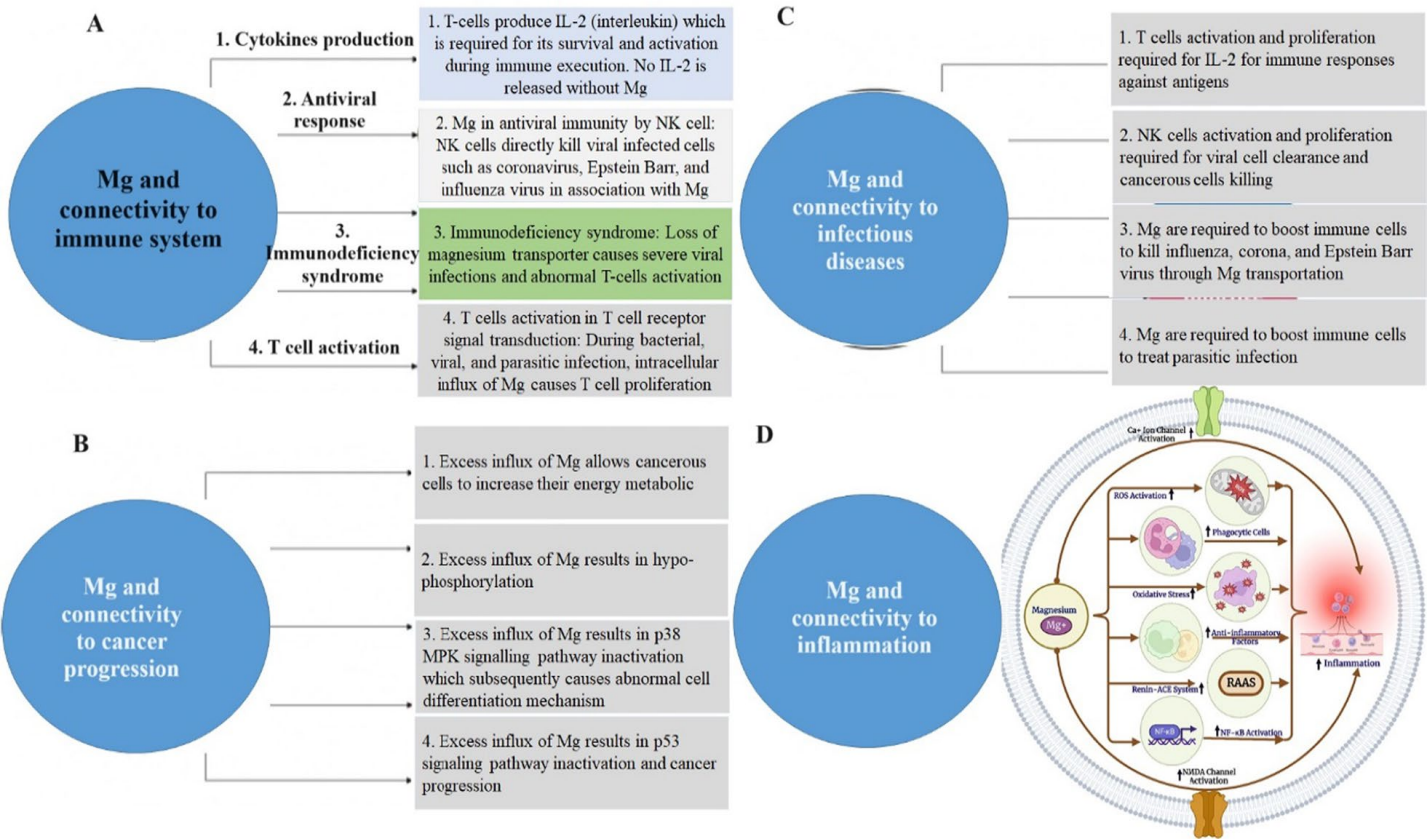
Role of magnesium in various physiological and pathological consequences. Mg is associated with immune response (**A**), cancer progression (**B**), infectious diseases (**C**), and inflammation (**D**). Inflammation is induced by magnesium depletion via numerous signaling mechanisms [40, 103]. *NMDA* N-methyl-D-aspartate, *RAAS* the renin–angiotensin–aldosterone system

**Fig. 4:**
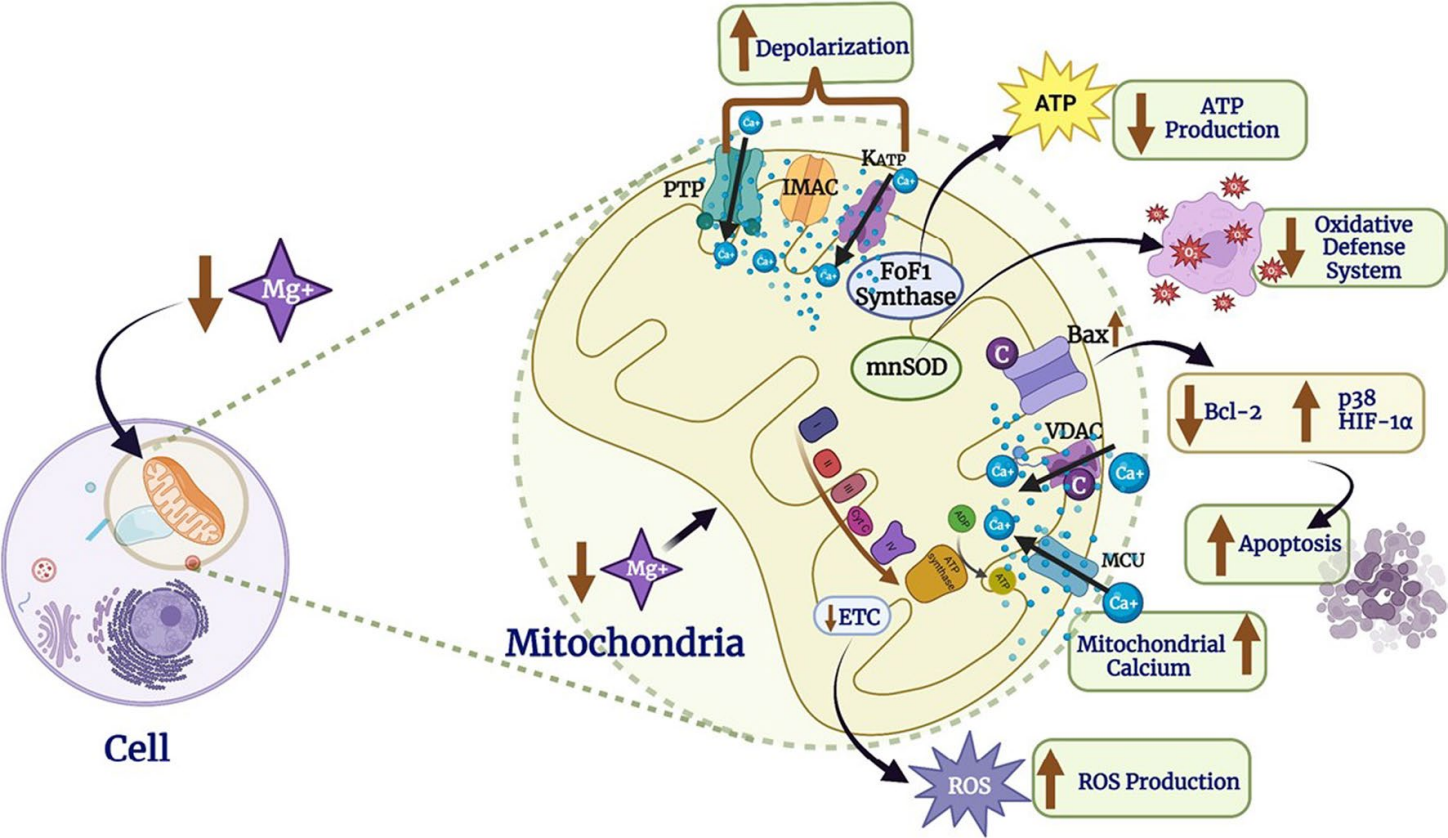
Intracellular Mg deprivation induces oxidative stress including malfunction in mitochondria [40]. *ATP* adenosine triphosphate, ETC electron transport chain, *F0F1-ATPases* membrane-bound ATP synthases, *IMAC* inner membrane anion channel, *KATP* ATP-sensitive K channel, *MnSOD* manganese superoxide dismutase, *MRS2* mitochondrial RNA splicing 2, *PTP* permeability transition pore, *ROS* reactive oxygen species, *VDAC* voltage-dependent anion channel

**Table 1:** Relevance of Mg deficiency in various pathological abnormalities as investigated in animal model and clinical trials

Biomarkers	Findings	Refs
↑ IL-1a, IL-6, NO, and VCAM	Mg deficiency promoted inflammation and angiogenesis. Low content of Mg provoked increased level of IL-1a, IL-6, NO, and VCAM	[82]
↑MCP-1, MT, RANTES	Decreased level of Mg in erythrocytes of atherosclerotic patients. Decreased NK cells cytotoxicity potential	[83]
Alkaline phosphatase	Reduction in dietary Mg by 50% resulted in reduced bone mineral content and the volume of distal femur	[84]
Elastin/collagen ratio	Long term Mg deficiency in diet results in cardiovascular risk in rats	[85]
Plasma IL-6, fibrinogen, and erythrocytic lysophosphatidylcholine	Long term Mg deficiency in diet of aging rats is related to high blood pressure, inflammation, and oxidative distress	[85,86]
CRP, IL-6, TNF-α R2, soluble VCAM-1	Dietary Mg connectivity to inflammatory biomarkers and endothelial dysfunction in post-menopausal women in a cohort study	[87]
IL-8, NF-kB	Potential interplay of NF-kB and PPARY in cultured human endothelial cells	[88]
IL-1, IL-6, and TNF-alpha	Dietary Mg deficiency was induced in rodents to execute inflammatory responses as evidenced with high levels of ILs in circulation	[89]
ROS, ↑ 8-hydroxy-deoxyguanine, and ↑ IL-1 and IL-6	Low Mg was related to aging, oxidative distress, atherosclerosis, and other vascular disorders	[90]
Markers not reported	Mg sulfate reduced asthma in patients not responding to conventional medicine and steroidal drugs. Similarly, 68% of children hospitalization was reduced by Mg sulfate	[91]
Markers not reported	Intravenous administration of Mg sulfate reduced acute asthmatic inflammation when not responded to the first-line treatment	[92]
IL-6, ↑ alpha2-macroglobulin and alpha1-acid glycoprotein and ↑ fibrinogen	Inflammatory responses in response to acute deficient Mg in rat	[93]
↓ IL-6, CRP, and NF-kB	Clinically, Mg was recommended in covid-19 infected patients	[95]
Serum Mg level	No positive correlation between Mg deficiency and covid-19 infected myocardial diseases	[97]
A comprehensive review report	Mg deficiency is linked to diabetes, heart failure, and other cardiac issues	[104]

*CRP* C-reactive protein, *IL* interleukin, *Mg* magnesium, *MCP-1* monocyte chemoattractant protein-1, *NF-kB* nuclear factor kappa-light-chain-enhancer of activated B cells, *NO* nitric oxide, *RANTES* regulated upon activation, normal T cell expressed and secreted, *ROS* reactive oxygen species, *TNF* tumor necrosis factor, *VCAM* vascular cell adhesion molecule

The updated version aligns with ethical guidelines and policies and includes sufficient reference to previous articles. The authors sincerely apologize for the errors. The errors do not affect the conclusion of the article.

Figures 3 and 4 and Table 1 have been updated in this correction and the original article [1] has been corrected.

## References

[1] Ashique S, Kumar S, Hussain A (2023). A narrative review on the role of magnesium in immune regulation, inflammation, infectious diseases, and cancer. J Health Popul Nutr.

[2] Liu M, Dudley SC (2020). Magnesium, oxidative stress, inflammation, and cardiovascular disease. Antioxidants.

[40] Liu M, Dudley SC (2020). Magnesium, Oxidative Stress, Inflammation, and Cardiovascular Disease. Antioxidants.

[103] Gupta AA, Shekatkar M, Raj AT, Kheur S (2020). Potential Role of Magnesium in Cancer Initiation and Progression. Pathol Oncol.

